# Exploring the factors and spatial patterns of national night cultural tourism consumption agglomeration zones in China

**DOI:** 10.1016/j.heliyon.2024.e24132

**Published:** 2024-01-08

**Authors:** Shanmei Xiong, Hui Wang, Zhenwei Liao, Rahmat Hashim

**Affiliations:** a School of Economics and Management, Nanchang Institute of Science and Technology, Nanchang, Jiangxi, China; b School of Hospitality, Tourism & Events, Faculty of Social Sciences & Leisure Management, Taylor's University Malaysia, Subang Jaya, 47500, Malaysia; c Faculty of Economics and Management, National University of Malaysia, Malaysia

**Keywords:** Night culture and tourism consumption agglomeration zone, Spatial pattern, Influencing factors, GIS

## Abstract

This study is based on the theory of spatial structure and uses the geographic information system's (GISs) spatial analysis technology to investigate the spatial distribution characteristics and influencing factors of 243 national night cultural and tourism consumption agglomeration zones (NNCTCAZs) in China. Furthermore, this study employs various analytical methods, including the nearest index, geographic concentration index, imbalance index, nuclear density analysis, buffer analysis and geographic detector method. The results reveal that NNCTCAZs exhibit an imbalanced spatial distribution, with a predominant concentration in the east and southwest regions of China. Furthermore, the ‘core-edge’ structure of this distribution is discernible. The spatial distribution density of NNCTCAZs is uneven, with high-density areas primarily located in the Yangtze River Delta, Pearl River Delta and Sichuan–Chongqing regions. This distribution pattern exhibits the characteristics of being progressive, that is, strong in the east and west and having small agglomerations with large dispersion. In addition, these areas are mainly concentrated in the central regions of cities and the surrounding areas of popular tourist attractions. The spatial layout of NNCTCAZs is mainly influenced by the level of social development, the tourism industry and regional gross domestic product (GDP), which are considered the core determinants. Furthermore, the development level of traffic conditions plays a crucial role in shaping the spatial layout, whereas the impact of the cultural environment and economic conditions is comparatively less pronounced.

## 1 Introduction

The pandemic has dramatically affected the development of the tourism industry and posed unprecedented difficulties for the industry. The pandemic's recurring nature has made the tourism market's recovery and the future of the industry practically impossible [1]. Against this background, the city needs to inject new vigour and create more re-employment opportunities for residents. Olt et al. argued that nighttime tourism is the factor most directly associated with the economic growth of nighttime consumption in cities [2]. Roberts et al. pointed out that many European cities are keen to commercialise through the development of a ‘nighttime economy’, with government authorities, the tourism industry and the hospitality sector positioning nighttime cities as recreational and escapist (work stress) destinations [3].

The nighttime economy is increasingly contributing to the economies of cities, regions and countries worldwide [4], thus serving as a powerful tool for enhancing the vitality and competitiveness of cities in terms of providing diverse cultural experiences, stimulating consumption and creating jobs [1,4]. For example, the nighttime economy is reported to be the fifth largest industry in the UK, accounting for at least 8 % of UK employment and generating £60 billion in annual revenue [5]. In China, cities such as Beijing, Shanghai, Chengdu and Wuhan lead the development of nighttime tourism due to their rich nighttime attractions. In Beijing, Wangfujing recorded >1 million passengers at its peak at night. The unique night scene culture experience, especially the garden night scene culture, is more and more favoured by tourists [6]. Wang et al. (2023) stated that night tourism (1) is provided to tourists, residents and office workers; (2) occurs in the city and (3) has strong authenticity and cultural attributes. Night tourism has sub-categories such as city night scenes, night markets, night festivals, night formative arts, music festivals, commercial streets, camps, bars and 24-h museums [7].

In August 2019, the General Office of the State Council issued several Opinions on the Further Stimulating the Potential of Cultural and Tourism Consumption, calling for the steady promotion of culture and tourism consumption and explicitly mentioning the development of the holiday economy and the night economy and the development of catering, shopping, performances and other nighttime service activities under the premise of ensuring safety and not affecting the neighbouring residents. In early July 2021, the Ministry of Culture and Tourism again issued a notice specifying the selection and construction of >200 national night cultural and tourism consumption agglomeration zones (NNCTCAZs) in batches [8]. Subsequently, ‘promoting consumption’ and ‘prospering the night economy’ have become the themes. The creation of nighttime consumption scenes, the supply of nighttime cultural tourism products, the creation of nighttime business districts, the lighting of urban nightscapes, transportation services and nighttime consumption check-in places have all become the main compositions of local policies.

NNCTCAZ refers to the regional characteristic culture as the core. NNCTCAZ relies on a particular landscape environment at night to implement integrated scene design and building at night. This approach integrates cultural and tourism business depth, product diversity, infrastructure, consumption environment, management operation mechanism and brand and market influence. The NNCTCAZs constitute a solid driving force in industrial cluster space with tourism consumption radiation [9,10].

The characteristics of NNCTCAZ are as follows: it operates from sunset to the next day and has a large volume and area, forming a sizeable commercial agglomeration area; it offers a range of high-end service industries such as tourism, participation, landscape and comprehensive night tourism products and services. Additionally, NNCTCAZ showcases the local diversity and unique cultural charm of the region through its cultural offerings [10].

The construction of NNCTCAZ has become an essential strategy for cities to stimulate the development of the nighttime economy and establish a robust domestic market [11]. Consequently, creating NNCTCAZs and strengthening the overall planning and layout of the nighttime economy have become priorities for all sectors of society.

Night tourism is an essential form of tourism, which can increase the time content of tourism and allow tourists to feel the different scenes of the city during the day (Huang & Wang, 2018). According to the China Tourism Research Institute (2019), 6–10 p.m. is the golden 4 h of night tourism and the peak period of tourism consumption [12]. Tourists tend to concentrate on one or several prime hours at night, whereas there are relatively few tourists during other hours. Owing to the limitations of insufficient time for nighttime tourism, safety considerations, cost constraints and other conditions, urban nighttime tourism tends to be clustered around transport hubs, leisure and entertainment venues and accommodation facilities [13]. However, the spatial clustering of urban night tourism presents hidden dangers such as congestion, noise and occupation of residential living spaces [14].

Scholars have paid considerable attention to the development of nighttime tourism. However, existing studies have focused mainly on the impact of night tourism [15], night tourism products [16], night tourism experience [17] and nighttime tourism stakeholders [18] but placed relatively less emphasis on the nighttime consumption agglomerations that are being created in China. Therefore, analysing the spatial distribution pattern of NNCTCAZs and their influencing factors is of great theoretical and practical significance for strengthening the overall planning and layout of the nighttime economy, promoting regional economic development, meeting the diversified needs of tourists and improving residents’ living standards.

Therefore, the objectives of this study are (1) to analyse the distribution patterns and characteristics of NNCTCAZ in China and (2) to explore the factors affecting NNCTCAZs, which, by August 2022, had been announced in a list of 243 NNCTCAZs in China. For practical guidance, we must thoroughly understand the spatial distribution characteristics and influencing factors of NNCTCAZs.

The theory of spatial structure regards the interrelated components in a specific region as an organic functional body and considers that the spatial interaction and spatial location relationship between the organisms reflect the degree of geospatial agglomeration and agglomeration scale of the organisms [1]. The German researcher von Böventer first systematically analysed the spatial structure theory and studied the main factors for the differences in spatial structure [19]. At the beginning of the 20th century, western scholars began to introduce the spatial structure theory in the field of tourism [20]. Zhang et al. stated that the theory of the spatial structure of tourism can be seen as a spatial abstraction of the constituents of tourism, including different classification methods such as static tourism resources, dynamic tourists, regional consumer markets and point-like service facilities [1]. NNCTCAZ is the spatial carrier of nighttime cultural and tourism activities, with nighttime tourism being a crucial element of the nighttime economy. Accordingly, the spatial structure theory of geography can be applied to the study of NNCTCAZ to provide a reference for the overall development of nighttime tourism in tourist destinations.

Geographic information systems (GISs), in combination with remote sensing and mapping, play an essential role in all geographical and spatial aspects of natural resource development and management. These technologies provide powerful analytical and visualisation tools for describing, analysing and modelling the processes and functions of natural systems [21]. Thus, in recent years, GIS has been widely used in various fields to study the spatial distribution and factors affecting urban planning and tourism, urban agglomeration [22], leisure tourism industry [23], National Geopark [24] and rural tourism destinations [25]. Therefore, this study uses the GIS statistical analysis method to study the distribution of NNCTCAZs and analyse the influencing factors.

Taking China as the study area, based on the spatial structure theory and GIS spatial statistical analysis method, this study first divides China's 243 NNCTCAZs into 12 types and then performs a detailed classification and statistical analysis on their structural characteristics and spatial structure. The present study introduces the representative provinces with more NNCTCAZs and typical cases. Second, the spatial distribution characteristics of NNCTCAZ are systematically analysed through regional heterogeneity analysis, spatial balance analysis and spatial agglomeration analysis, and the overall distribution characteristics of NNCTCAZ are described. Third, this study draws on related studies to construct a system of 16 indicators of 5 major influencing factors and analyses the influence of these indicator elements on the spatial distribution pattern of NNCTCAZs. Finally, the results are analysed and discussed, providing a basis for developing effective planning and management policies for NNCTCAZs.

## 2 Literature review

### 2.1 Night economy

Research on the night economy in foreign countries began in the 1970s, with the British exploring ways to revitalise urban economies. In China, the nighttime economy started in the early 1990s and has developed from the early lighted night market into a diversified consumer market, including catering, tourism, shopping, entertainment, sports, exhibitions and performances [26]. With the gradual deepening of the integration of culture and tourism, tapping the ‘nighttime economy’ has become a focus of attention in the industry. Scholars from various countries have studied different aspects of the night economy, such as its characteristics [9,27], function and influence [2,3,[28], [29], [30]] and night city management [13,31]. Numerous studies have focused on the social impact of the night economy and related topics from the perspectives of urban science and sociology.

The night economy has developed differently in various regions due to variations in the social environment and economic stages. Consequently, the focus and content of research on the night economy differ across different countries. In China, urban night tourism is the current hotspot of research on the night economy.

### 2.2 Night tourism

The emergence of night tourism within the night economy results from the changing consumer demand of tourists and the increasing leisure demand of urban residents. Night tourism fills the gap in the demand for night activities, enriches the evening lives of tourists and improves residents' quality of life [32]. Song et al. (2020) defined the concept of night tourism as different activities conducted by tourists and residents from sunset to midnight in tourist destinations [33]. According to Li (2021), China's night tourism has formed four typical development models: performance, landscape, participation and comprehensive [6]. The performance type mainly focuses on performing arts activities, including live and folk performances. The landscape type mainly focuses on watching the night landscape; the participation type mainly focuses on tasting snacks, shopping and entertainment; and the comprehensive type focuses on designing comprehensive night tour tourism routes [6].

Nighttime is often perceived as a transgressive space without standard rules and norms, and the unique attraction of the ‘night’ shapes the city's distinctive night landscape [18]. At the same time, urban architecture, heritage landscapes, unique communities and other illuminated landscapes are essential for city night tourism. Light and shadow technology has become essential in developing night tourism in many cities [34,35]. Scholars have argued that the use of night lighting to create urban spatial landscapes has a revitalising effect and can improve function in public spaces, and that light art and lighting strategies can be used to promote the conservation and presentation of architectural or natural sites [36].

With the rapid growth of tourism in cities, many tours that were initially daytime have extended their opening hours to late nights. Urban facilities with extended opening hours, including cultural venues such as museums and zoos, also play a crucial role in nighttime tourism by offering unique experiences [32,37]. Night markets, industrial sites, local culture and leisure and entertainment venues are essential for nighttime tourism [16,38,39]. For example, night markets in Taiwan and Thailand are popular among residents and foreign tourists because they offer local specialties, products, entertainment and leisure activities. In addition, nighttime performances and night festivals are crucial vehicles for nighttime tourism. To attract tourists, many national and regional cities seize the opportunity to organise late-night festivals and events with special night shows, museum nights and events; examples include the Pink Night Festival in Italy, the Newt Blanche in Paris and the Marianne Night Parade in France [40,41].

Extensive studies in the field have promoted the strategic development of night tourism, improved the attractiveness of night tourism activities to tourists and residents and enriched the content of night tourism activities. However, the literature analysis suggests the need to focus more on the nighttime cultural tourism consumption agglomeration area, an essential new engine for promoting the nighttime economy and developing nighttime tourism.

Although the development of night tourism began later in China than in other countries, the country's progress has been impressive. Currently, China is actively creating NNCTCAZs focused on the night economy. However, studies have not considered the macro-levels of night tourism consumption, its spatial structure and influencing factors. This lack of overall understanding of the spatial layout of the night economy makes it challenging to promote the development of NNCTCAZs and the optimisation of the spatial pattern of the night economy.

## 3 Data sources and study methods

### 3.1 Data source

The data sample used in this study is based on 120 and 123 NNCTCAZs, as announced by the Ministry of Culture and Tourism, PRC. The sample comprises the first and second batches of data published in 2021 and 2022, respectively, covering 31 provinces (municipalities, autonomous regions and provinces) excluding data from Hong Kong, Macao and Taiwan (https://www.mct.gov.cn). This study uses Google Maps to determine the geographical coordinates of each night's culture and tourism consumption cluster area. Furthermore, the current study uses ArcGIS software to establish the spatial distribution database of China's NNCTCAZs. The regional economic and social development data have been obtained from the China Statistical Yearbook [42]. The data on 5A scenic spots and the national tourist resorts (by the end of 2022) have been acquired from the official website of the Ministry of Culture and Tourism. At the same time, the night light remote sensing data have been obtained from the China Artificial Night Light Data Set of the National Qinghai–Tibet Plateau Scientific Data Centre (http://data.tpdc.ac.cn). Finally, basic geographic data, including administrative boundaries and road and railway networks, are obtained mainly from the National Geographic Information Resources Catalogue Service System (https://www.webmap.cn/main.do?method=index).

### 3.2 Research technique

#### 3.2.1 Nearest neighbour index

On the map, NNCTCAZs during the night appear as dots and exhibit three spatial distribution types: clustered, uniform or evenly distributed and random. The nearest neighbour index (nearest neighbour ratio, NNR) of the point element reflects the spatial distribution type. NNR is calculated as the ratio of the actual nearest neighbour distance to the theoretical nearest neighbour distance (i.e. the theoretical value at random distribution). The calculation formula is as follows [43]:

$$r_{E}=1/\sqrt{n/A}=1/\sqrt{D}$$

$$R={\bar{r}}_{i}{/r}_{E}=2\sqrt{D}{\bar{r}}_{i}$$

where R denotes the nearest neighbour exponent, r_i_ denotes the actual nearest neighbour distance, *r*_*E*_ denotes the theoretical nearest neighbour distance, n denotes the number of NNCTCAZ, A denotes the regional area and *D* denotes the number of NNCTCAZs. When R < 1, the spatial distribution pattern is clustered; when R = 1, the pattern is random; and when R > 1, the distribution pattern is uniform.

#### 3.2.2 Geographic concentration index

The geographic concentration index indicates the degree of agglomeration in the spatial distribution of the research objects. The index is calculated as follows [44]:

$$G=100\%\times \sqrt{\sum_{i=1}^{t}{\left(\frac{x_{i}}{T}\right)}^{2}}$$

where G denotes the geographic concentration index of NNCTCAZ; x_i_ denotes the distribution number of the consumption clusters in the district city i; T denotes the total number of NNCTCAZ in China; t denotes the total number of Chinese provinces, autonomous regions and municipalities directly under central government and G_0_ is set as an equilibrium index denoting an even distribution of the geographic concentration index across all provinces and cities in China. G > G_0_ indicates a concentrated distribution, whereas G < G_0_ indicates a scattered distribution.

#### 3.2.3 Lorentz curve

The Lorentz curve indicates the degree of equilibrium of the spatial distribution of geographical elements. This study uses the bending degree of the Lorentz non-equilibrium index observed across various provinces and cities to assess the distribution of NNCTCAZs within different regions of China. The calculation formula is as follows [45]:

$$S=\frac{\sum_{i=1}^{n}Y_{i}-50\left(n+1\right)}{100n-50\left(n+1\right)}$$

where S denotes the Lorentz non-equilibrium index; *n* denotes the number of provinces and cities and *Y*_*i*_ is the cumulative percentage of the *i*th place in the ranking of the ratio of the number of NNCTCAZ to the total number of consumption clusters in each province, from largest to smallest. The value of S, which ranges from 0 to 1, indicates the level of concentration in the distribution of NNCTCAZs. The closer the S is to 0, the more balanced the distribution of NNCTCAZs is.

#### 3.2.4 Nuclear density estimation

Nuclear density estimation, which indicates the spatial distribution density of regional elements, can be used to examine the morphological characteristics of the spatial distribution of geographical elements in regions. This study uses the nuclear density estimation method to analyse the density of the spatial distribution of NNCTCAZs. The calculation formula is as follows [46]:

$$f\left(x\right)=\frac{1}{\text{Th}}\sum_{i=1}^{T}k\left(\frac{x-X_{i}}{h}\right)$$

where f (x) denotes the core density estimate; T denotes the total number of NNCTCAZs; h denotes the search radius and x denotes the location of the NNCTCAZ to be estimated. X_i_ denotes the location of the ith NNCTCAZ within a spatial range, with x as the centre and h as the radius. A larger kernel density estimate indicates a higher density of points and a higher probability of geographic events.

#### 3.2.5 Geographic detectors

A geodetector is a set of statistical methods used to detect the spatial differentiation of geographical elements and identify their comprehensive determinants [47]. This study considers numerous factors affecting the spatial pattern of the NNCTCAZ, making it challenging to fully meet the assumptions of the traditional statistical analysis methods, which could potentially affect the evaluation results. Geodetectors require fewer assumptions and can effectively overcome the limitations of treating category variables in traditional statistical analysis methods [48]. Accordingly, the geographical detection method is used in this study to analyse the effect of various factors on the spatial distribution of NNTCCZs. The calculation formula is as follows:

$$q=1-\frac{1}{N\sigma^{2}}\sum_{h=1}^{L}N_{h}\sigma_{h}^{2}\text{,}$$

where q denotes the influence of the spatial distribution of NNTCCZs; *L* denotes the spatial distribution quantity of NNCTCAZs; *N* and *N*_*h*_ denote the number of cells in the entire region and the secondary regions, respectively; *σ*^2^ and $\sigma_{h}^{2}$ denote the variance of the dependent variable in the entire region and the secondary regions, respectively. This study considers the number of NNCTCAZs as the dependent variable and detection factors such as economic development as the independent variables. The value interval of q is [0,1]; the closer the q value is to 0, the less influential the factor is in driving the spatial distribution of these agglomeration areas.

## 4 Spatial analysis of NNCTCAZs

### 4.1 Type and structural characteristics of NNCTCAZs

#### *4.1.1* *Type and structure of* NNCTCAZs

In the present study, 243 NNCTCAZs were studied through various related sources (www.cslab.sdu.edu.cn). Based on the similarity and heterogeneity, the NNCTCAZs were classified into 12 categories including historical and cultural blocks (Nanjing Confucius temple–beside Qinhuai Scenic Belt); cultural and tourism leisure commercial districts (Chengdu Chunxi Road); cultural and tourism towns (Suzhou Zhouzhuang Town); scenic areas (Yangzhou West Lake); historical and cultural cities (Yunnan Lijiang); cultural industrial parks (Shenzhen Shekou Coastal Cultural Creative Blocks); tourist resorts (Guangzhou Mayor Long Resort); theme park types (Beijing Chaoyang Happy Valley); tour performing arts (the romantic show of Sanya); sports, culture, commerce and tourism integration (West Bank Art Museum Avenue, Xuhui District, Shanghai); Industrial Heritage Creative (798-751 Art Block, Chaoyang District, Beijing) and urban riverside leisure (Beibei District, Chongqing).

**Fig. 1:**
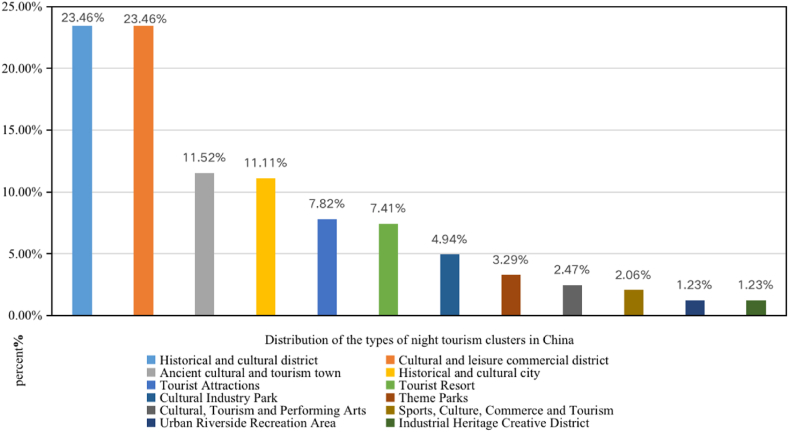
Types and structure of NNCTCAZs.

As shown in Fig. 1, the historical and cultural blocks and cultural tourism and leisure commercial districts constituted the highest proportion (23.4 %), followed by the cultural tourism ancient town, accounting for 11.52 %. Historical and cultural cities constituted 11.11 %, and the other types constituted <10 % (Fig. 1).

#### *4.1.2* *Spatial structure of various types of* NNCTCAZs

Table 1 presents the spatial structure characteristics of various types of NNCTCAZs, considering the seven geographical divisions. Historical and cultural blocks and cultural and tourism leisure commercial districts constituted a large and relatively stable proportion in each region. Furthermore, NNCTCAZs differed in their spatial structure characteristics.

**Table 1:** Spatial structure of NNCTCAZs by type %.

Type	North China Region	Northeast China	Eastern China Region	China Central Region	South China Region	Southwest Region	Northwest China
Historical and cultural district	21.88	27.27	30.77	11.54	20	19.57	24
Cultural and leisure commercial district	15.63	36.36	23.08	26.92	24	15.22	40
Ancient cultural and tourism town	9.38	9.09	10.26	7.69	12	21.74	4
Historical and cultural city	9.38	0	8.97	23.08	8	15.22	8
Tourist attractions	12.5	9.09	6.41	15.38	0	6.52	8
Tourist resort	3.13	0	6.41	11.54	12	10.87	4
Cultural industry park	6.25	0	3.85	3.85	8	2.17	12
Theme parks	3.13	0	5.13	0	8	2.17	0
Cultural, tourism and performing arts	3.13	0	3.85	0	4	2.17	0
Sports, culture, commerce and tourism	9.38	9.09	1.28	0	0	0	0
Urban riverside recreation area	3.13	0	0	0	4	2.17	0
Industrial heritage creative district	3.13	9.09	0	0	0	2.17	0

Regarding provincial distribution, historical and cultural blocks are primarily located in Jiangsu, Jiangxi, Guangxi, Fujian, Zhejiang and Chongqing. The Confucius temple–Qinhuai Scenic Belt Project in Nanjing, Jiangsu, has been a night consumption cluster in China since ancient times. This project, with the Confucius temple as the centre and the Qinhuai River as the axis, covers an area of 2.94 km^2^ and integrates nighttime sightseeing, dining, entertainment, shopping and other activities.

Cultural, tourism and leisure commercial blocks are primarily concentrated in Hunan, Beijing, Fujian, Guangdong, Jiangxi, Shanghai and Xinjiang provinces. In 2021, Changsha's night economy ranked second in the country and has become a popular destination for young people due to its reputation as a ‘web celebrity city’. The city has witnessed considerable growth in its night economy in recent years and is widely regarded as a ‘city of happiness’ among its youthful population.

The province with the highest distribution of ancient towns is Guizhou, with five towns being the most distributed. Examples of these towns include the Qingyan Ancient Town Scenic Spot in Guiyang City, Guizhou Province; Danzhai Wanda Town in Qiandongnan Miao and Dong Autonomous Prefecture; Maotai Liquor in Zunyi City; Xijiang Qianhu Miao Village in Qiandongnan Miao and Dong Autonomous Prefecture and Libo Ancient Town in Qiannan Buyie and Miao Autonomous Prefecture. These towns are known for their ethnic minority customs and unique ancient buildings, making them popular tourist destinations.

Famous historical and cultural cities are mainly distributed in Shandong, Shanxi, Sichuan and Yunnan provinces. Lijiang City in Yunnan and Pingyao in Shanxi, the world's cultural heritage sites, are major tourist attractions. With the idea of integrating cultural and tourism elements, efforts have been directed towards the protection and utilisation of these historical and cultural cities. By focusing on the ancient cities and actively promoting their business radiation and influence, these historical and cultural cities have been driving the development of night economies. The distribution of tourist attractions in the provinces, cities and autonomous regions is relatively balanced. Tourist resorts are primarily distributed in Shandong, Sichuan and Yunnan provinces. The distribution of cultural industrial parks is limited, with Guangdong and Shanxi provinces being the primary locations. Foshan Creative Industrial Park in Foshan City, Guangdong Province, is the most prominent representative of this type of park. This park has been recognised as one of the top 10 popular night markets among Chinese tourists, with a characteristic pedestrian street in Guangdong Province.

The distribution of theme parks is concentrated in Zhejiang, Jiangsu, Beijing, Guangdong, Anhui and Hainan. For example, Hangzhou Songcheng in Zhejiang and Happy Valley in Beijing have witnessed the expansion of night consumption activities as an essential component in the development of holiday tourism and achieved better transformation and development by continuously improving the surrounding facilities and offering diverse experiences such as an immense night landscape, night entertainment, night catering, shopping and accommodation. Tourism and performing arts are prevalent in Shanghai, Shandong, Chongqing, Beijing, Jiangxi and Hainan provinces. By promoting the performing arts industry, these regions have witnessed increased consumption of nighttime cultural tourism.

The integration of sports, business and tourism is mainly observed in areas such as West Bund Art Museum Avenue, Xuhui District, Shanghai; Huaxi Live, Wukesong, Haidian District, Beijing; Fudi Sports, Business and Tourism Block, Langfang City, Hebei Province; Binhai Cultural Centre of Tianjin Binhai New Area and Qiile Complex in Changchun, Jilin Province. However, there are only three urban riverside leisure areas and industrial heritage creativity areas. Urban riverside leisure areas are Chongqing Beibei District, Binjiang Leisure Area, Beijing Chaoyang District, Liangma River-style waterfront in Nanning and Guangxi Yongjiang south bank area. Furthermore, industrial heritage ideas are mainly observed in Chongqing, Beijing and Liaoning.

### 4.2 Spatial distribution characteristics of NNCTCAZs

#### 4.2.1 Regional differentiation characteristics

**Fig. 2:**
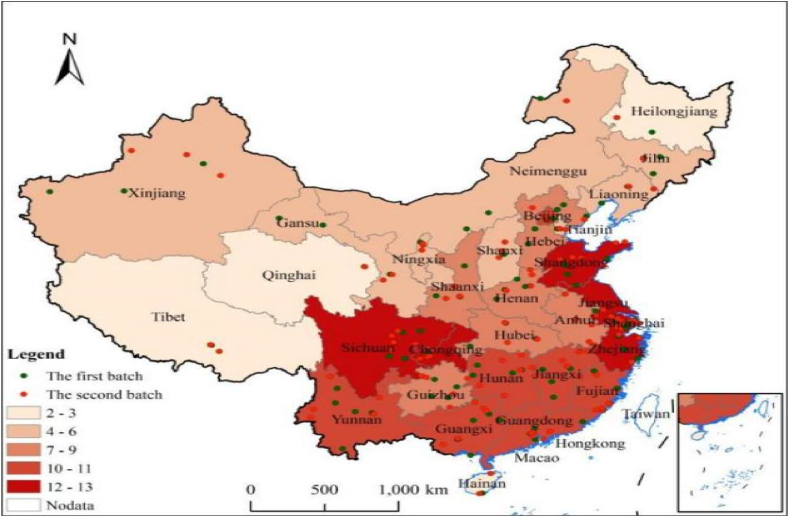
Overall spatial distribution of NNCTCAZs.

Fig. 2 shows the overall spatial distribution of NNCTCAZs. Data analysis using ArcGIS 10.5 software showed that the average nearest neighbour index of NNCTCAZs was 0.458, with a Z-value of −16.160. The significance test, with a P-value of 0.000, indicates that the spatial distribution of NNCTCAZs in the entire country is statistically significant.

**Fig. 3:**
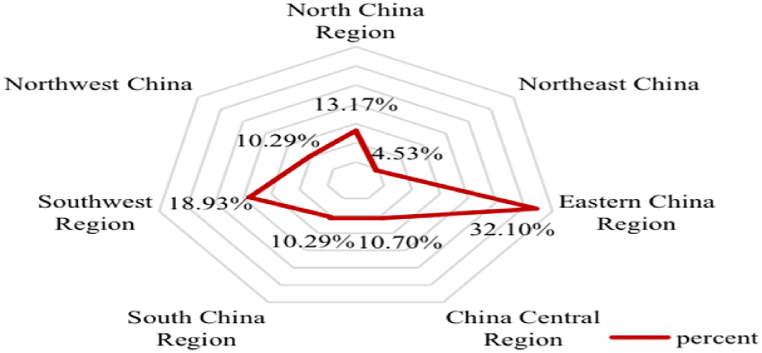
Spatial distribution of NNCTCAZs in seven geographical divisions.

Table 2 shows the number and proportion of NNCTCAZs in China according to three major geographical divisions and seven major geographical divisions. NNCTCAZs were distributed across the eastern, central and western regions, accounting for 41.56 %, 23.05 % and 35.39 %, respectively. The distribution pattern of these areas can be described as ‘a strong east, a thriving west and a progressive centre’. Across the seven major geographical divisions in China, the proportions of NNCTCAZs in Northeast China, North China, East China, Central China, South China, Southwest China and Northwest China were 4.53 %, 13.17 %, 32.10 %, 10.70 %, 10.29 %, 18.93 % and 10.29 %, respectively. The highest proportion of 32.10 % was observed in East China, whereas the lowest proportion of only 4.53 % was found in Northeast China (Table 2, Fig. 3).

**Table 2:** Spatial distribution of the three major sub-regions and the seven major sub-regions of the NNCTCAZs.

Region	Provinces, cities, autonomous regions	Number	%		Provinces, cities, autonomous regions	Number	%
Eastern	Shanghai	12	4.94	Northeast China	Heilongjiang	2	0.82
	Jiangsu	12	4.94		Jilin	4	1.65
	Zhejiang	12	4.94		Liaoning	5	2.06
	Shandong	12	4.94		**Total**	**11**	**4.53**
	Beijing	11	4.53	North China Region	Beijing	11	4.53
	Fujian	11	4.53		Hebei	8	3.29
	Guangdong	11	4.53		Shanxi	5	2.06
	Hebei	8	3.29		Inner Mongolia	4	1.65
	Liaoning	5	2.06		Tianjin	4	1.65
	Tianjin	4	1.65		**Total**	**32**	**13.17**
	Hainan	3	1.23	Eastern China Region	Shanghai	12	4.94
	**Total**	**101**	**41.56**		Jiangsu	12	4.94
					Zhejiang	12	4.94
					Shandong	12	4.94
Central	Jiangxi	11	4.53		Fujian	11	4.53
	Hunan	10	4.12		Jiangxi	11	4.53
	Henan	9	3.70		Anhui	8	3.29
	Anhui	8	3.29		**Total**	**78**	**32.10**
	Hubei	7	2.88	China Central Region	Hunan	10	4.12
	Shanxi	5	2.06		Henan	9	3.70
	Jilin	4	1.65		Hubei	7	2.88
	Heilongjiang	2	0.82		**Total**	**26**	**10.70**
	**Total**	**56**	**23.05**	South China Region	Guangdong	11	4.53
					Guangxi	11	4.53
					Hainan	3	1.23
Western	Sichuan	13	5.35		**Total**	**25**	**10.29**
	Chongqing	12	4.94	Northwest China	Shaanxi	8	3.29
	Guangxi	11	4.53		Xinjiang	6	2.47
	Yunnan	10	4.12		Gansu	5	2.06
	Guizhou	8	3.29		Ningxia	4	1.65
	Shaanxi	8	3.29		Qinghai	2	0.82
	Xinjiang	8	3.29		**Total**	**25**	**10.29**
	Gansu	6	2.47	Southwest China	Sichuan	13	5.35

Regarding the number of provinces, the first echelon comprises Sichuan, Shanghai, Chongqing, Jiangsu and Shandong, with 12–13 nightly tourism clusters. The second tier includes Beijing, Fujian, Guangdong, Guangxi, Jiangxi, Hunan and Yunnan, with 10–11 nightly tourism clusters. Hainan (3), Tibet (3), Qinghai (2), Heilongjiang (2) and four provinces and autonomous regions are situated at the end of the country. This selection of provinces is balanced and reflects the strengths of regional cultural and economic development. Additionally, it aligns well with the national and regional development strategies of Beijing–Tianjin–Hebei, the Yangtze River Delta, the Pearl River Delta, the Chengdu–Chongqing Economic Circle and local cultural and tourism resources. The interprovincial spatial distribution of NNCTCAZs varies significantly. NNCTCAZs are highly concentrated in the east but less in the west. Additionally, the distribution of NNCTCAZs is characterised by a few large clusters and many small clusters. Furthermore, there needs to be more NNCTCAZs among provinces, municipalities and autonomous regions because economically developed areas have relatively more NNCTCAZs than underdeveloped areas.

#### 4.2.2 Spatial equilibrium analysis

Table 3 presents the number, percentage and cumulative percentage of NNCTCAZs in all provinces, municipalities and autonomous regions.

**Table 3:** Distribution of NNCTCAZs by province, city and autonomous region in China.

Serial number	Provinces, cities, autonomous regions	Number	%	Cumulative percentage
1	Sichuan	13	5.35	5.35
2	Shanghai	12	4.94	10.29
3	Jiangsu	12	4.94	15.23
4	Zhejiang	12	4.94	20.16
5	Shandong	12	4.94	25.10
6	Chongqing	12	4.94	30.04
7	Beijing	11	4.53	34.57
8	Fujian	11	4.53	39.09
9	Jiangxi	11	4.53	43.62
10	Guangdong	11	4.53	48.15
11	Guangxi	11	4.53	52.68
12	Yunnan	10	4.12	56.79
13	Hunan	10	4.12	60.91
14	Henan	9	3.70	64.61
15	Anhui	8	3.29	67.90
16	Hebei	8	3.29	71.19
17	Guizhou	8	3.29	74.49
18	Shaanxi	8	3.29	77.78
19	Hubei	7	2.88	80.66
20	Xinjiang	6	2.47	83.13
21	Shanxi	5	2.06	85.19
22	Liaoning	5	2.06	87.24
23	Gansu	5	2.06	89.30
24	Inner Mongolia	4	1.65	90.95
25	Tianjin	4	1.65	92.59
26	Jilin	4	1.65	94.24
27	Ningxia	4	1.65	95.88
28	Hainan	3	1.23	97.12
29	Tibet	3	1.23	98.35
30	Qinghai	2	0.82	99.18
31	Heilongjiang	2	0.82	100.00
	Total	243	100	

The geographic concentration index is a measure of the concentration of the spatial distribution of NNCTCAZs. In the current study, the geographic concentration index of NNCTCAZ was determined to be 19.65. This value is compared to the actual geographic concentration index, which is 17.96 and is calculated assuming that the 243 NNCTCAZs are evenly distributed in 31 provinces, municipalities and autonomous regions in China. The actual calculated geographic concentration index value is greater than the geographic concentration index value calculated under assumptions, suggesting that the spatial distribution of NNCTCAZs in China is relatively concentrated among provinces, municipalities and autonomous regions. However, the geographic concentration index reflects the centralised distribution only at the macro-level and does not reveal the distribution pattern within provinces and autonomous regions.

**Fig. 4:**
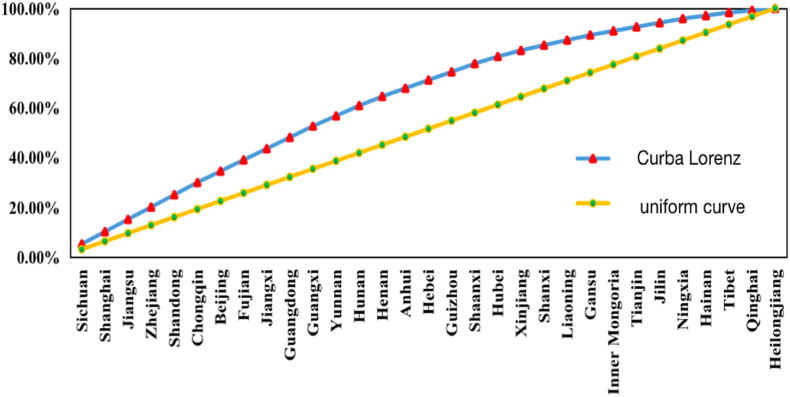
Spatially distributed Lorenz curves.

Furthermore, the imbalance index explains the equilibrium degree of the distribution of night tourism clusters in the provinces and cities. The imbalance index of NNCTCAZs is calculated at 0.26, which is relatively low. The Lorentz curve reflects the distribution equilibrium of the NNCTCAZs in all provinces and cities in China. The curve deviates from the uniform distribution line and presents convex characteristics (Fig. 4). Sichuan, Shanghai, Jiangsu, Zhejiang, Shandong, Chongqing, Beijing, Fujian, Jiangxi, Guangdong and the Guangxi Zhuang Autonomous Region constituted 52.68 % of all provinces, cities and autonomous regions in China. The results highlight the imbalance in the distribution of these clusters within China's provinces, cities and autonomous regions.

#### 4.2.3 Spatial agglomeration analysis

**Fig. 5:**
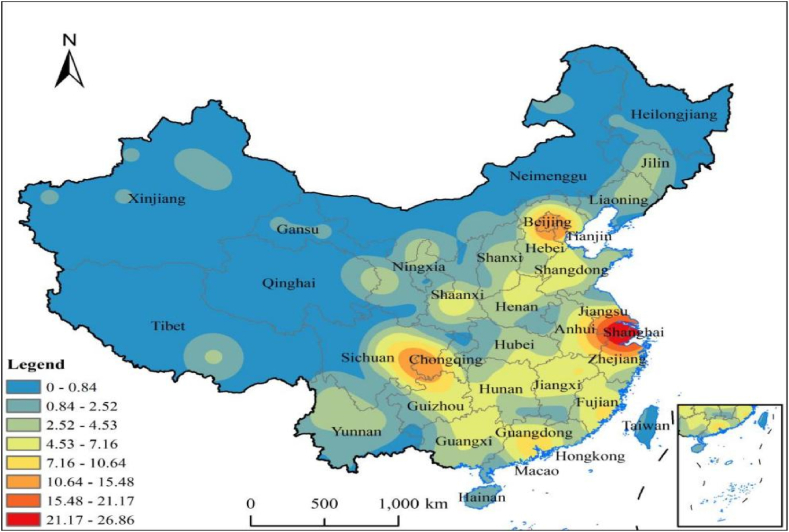
Nucleus density distribution of NNCTCAZs in China.

This study uses ArcGIS 10.5 software to analyse the nuclear density of NNCTCAZs and determine their spatial agglomeration direction. As shown in Fig. 5, NNCTCAZs in all provinces, municipalities and autonomous regions of China exhibit a clustering pattern characterised by ‘dense in the east, sparse in the west, with single cores and multiple centres’. This pattern indicates the formation of a high-density core area, two sub-advanced core areas and three three-level core areas. The high-density core area is in the Yangtze River Delta, with a nuclear density value ranging from 21.27 to 26.86. This core area is centred around Shanghai and extends to neighbouring areas, including the intersection of Jiangsu, Anhui and Zhejiang. The distribution of the clusters in this area follows a semi-ring-circle structure with a decreasing density value. The nuclear density values of the two secondary core areas range from 10.64 to 15.48; one of them is in eastern Sichuan, Chongqing and northern Guizhou, whereas the other, with Beijing as the core, is in the north of Hebei, southwest of Tianjin radiation. The three three-level core areas are single-core distribution areas formed by Guangdong, Fujian and Jiangxi.

Furthermore, NNCTCAZs in the southeast and southwest of China are extensively concentrated but exhibit a sporadic distribution in the northwest. In summary, NNCTCAZs are mainly distributed in urban centres with good economic foundations, dense populations, well-developed tertiary industries and famous tourist attractions. The current study highlights a ‘core-edge’ structure in the distribution of NNCTCAZs. The results suggest a close association between the concentration of these clusters and economic, social and tourism development.

## 5 Factors influencing the spatial distribution of NNCTCAZ

### 5.1 Selection of influencing factors

The present study builds upon previous research on the night economy and leisure tourism space conducted by Luo and Li (2021), Liao et al. (2022) and Zhao and Liu (2021) [10,23,25]. An index evaluation system is established by considering the unique characteristics of the NNCTCAZs and the relevant features of night tourism. This system comprises five significant influencing factors: economic development level, social development level, tourism industry development level, traffic conditions and cultural environment. The system covers various aspects such as economic foundation, population size, living standards, the number of employees in the tertiary industry, transportation accessibility, cultural development, the tourism market and service facilities. The selection is based on scientific validity, relevance and data accessibility.

The indicators for the economic development level consider the correlation between the local economic base and the agglomeration of leisure business forms. Furthermore, upgrading consumer demand and improving consumption levels can promote the establishment of a night economy and night tourism consumption. Factors such as regional (gross domestic product) GDP and the night lighting index can serve as suitable indicators to measure these aspects. Accordingly, this study considers the regional GDP, night light index, per capita disposable income of urban and rural residents, per capita living consumption expenditure of urban and rural residents and proportion of the tertiary industry as evaluation indicators. Indicators assessing the level of social development consider that NNCTCAZs cater to the residents and foreign tourists, thereby addressing their recreational and consumption needs. Theoretically, the distribution of the NNCTCAZs strongly correlates with population size and the employment of the tertiary industry. Accordingly, the number of permanent residents at the end of the year and the number of employees in the tertiary industry in urban, non-private units are used as evaluation indicators.

Indicators assessing the level of development of the tourism industry consider that overnight tourists are the main tourists of NNCTCAZs. The spatial distribution of such areas is closely related to the level of local tourism development, the availability of tourism resources and the quality of service facilities. Accordingly, the total tourism revenue, number of tourists, number of 5A scenic spots and number of national tourist resorts and star-rated hotels are used as evaluation indicators. Indicators used to assess traffic conditions mainly consider that night tourism is limited by time and space and places high demands on traffic accessibility and transportation capacity. Accordingly, highway density, railway density and annual passenger volume are evaluation indicators. Indicators assessing cultural environmental factors consider the cultural aspects of night tourism, where cultural products of night tourism mainly rely on local cultural resources. Accordingly, the number of cultural performance groups and several national intangible cultural heritage sites are used as evaluation indicators.

### 5.2 Analysis of influencing factors

In the current study, the geographical detector method is used to analyse factors determining the spatial distribution of NNCTCAZs within provinces, municipalities and autonomous regions of China. For traffic analysis, the buffer zone is used to examine the spatial effect of road and railway density on the tourism consumption accumulation area. Using the natural breakpoint method, the continuous variable is first divided into five categories in ArcGIS 10.5 software. Subsequently, the geographical detector method is utilised to determine factors affecting the spatial distribution pattern of NNCTCAZs. The results are presented in Table 4, where a low q value indicates a weak impact of the index factor, and a higher q value indicates a more significant influence.

**Table 4:** Factor detection results.

Factors of influence	Evaluation indicators	Influence q value	Mean value of influence q
Level of economic development	Regional GDP	0.765	0.343
	Nighttime lighting index	0.101	
	Per capita l disposable income of urban and rural residents	0.466	
	Per capita living consumption expenditure of urban and rural residents	0.179	
	Percentage of tertiary sector	0.206	
Level of social development	Number of resident population at year end	0.777	0.774
	Number of employees in the tertiary sector in the urban non-private sector	0.771	
Transportation conditions	Road density	0.577	0.6935
	Annual passenger capacity	0.810	
Cultural environment	Number of cultural performance groups	0.475	0.4255
	Number of national-level intangible cultural heritage	0.376	
Level of tourism industry development	Total tourism revenue	0.795	0.702
	Total tourist arrivals	0.723	
	Number of 5A scenic spots	0.674	
	National Resort District	0.719	
	Number of star-rated hotels	0.599	

The average q value for the influence of economic development level on the spatial arrangement of NNCTCAZs is 0.343. However, the regional GDP (q = 0.765) significantly impacts the spatial layout of NNCTCAZs. The analysis results indicate that the local economic development level has laid a solid foundation for the development of NNCTCAZs. The per capita disposable income of urban and rural residents (q = 0.466) has a strong effect, whereas the proportions of the tertiary industry in the index (q = 0.206) and the per capita living consumption expenditure of urban and rural residents (q = 0.179) are relatively weak. The urban economic development level is related to the market potential and development scale of the NNCTCAZs. Cities with high economic development levels can meet the basic requirements of water and electricity supply, traffic order control, market fire safety management and other aspects of night tourism, facilitating the establishment and development of NNCTCAZs.

The per capita disposable income of urban and rural residents considerably impacts residents' night consumption, with the premise of tourism being ‘money’ and ‘leisure’. Residents can satisfy their desire to consume only when they possess more disposable income. NNCTCAZs represent a tertiary industry cluster that comprises various forms such as entertainment, shopping, catering, culture and accommodation, and their spatial distribution is significantly affected by the development of the tertiary industry. Generally, under a high proportion of the tertiary industry, a city's service industry tends to be more developed, leading to an excellent supply of nighttime cultural and tourism products. The impact of the night light index (with a value of 0.101) on the spatial layout of NNCTCAZs is somewhat noticeable, but it is not particularly strong, consistent with Luo and Li's findings [10].

Overall, nighttime economic activity tends to benefit from adequate lighting, and areas with higher brightness levels at night often have higher economic activity during those hours. Despite being the brightest city on the night light map in the Beijing–Tianjin–Hebei region, Tianjin has only four NNCTCAZs and is ranked lower than other cities. This may be because some cities have a well-developed secondary industry and high lighting brightness levels but not necessarily a robust cultural tourism economy.

Social development is the core factor affecting the spatial layout, with the average q value reaching 0.774. Furthermore, the permanent population (q = 0.777) and the number of employees in the third industry in urban private units (q = 0.771) have a similar influence on the spatial layout of NNCTCAZs. This is because the development of the nighttime economy requires the extension of business hours and an increase in the number of service personnel at night. Therefore, areas with a high concentration of night tourism consumption tend to rely heavily on a dense labour market, with a sufficient pool of potential employees available to support the industry. In addition, strong consumer demand for the NNCTCAZs promotes the rapid development of the nighttime cultural tourism economy.

Population size plays a significant role in determining the level of demand for the NNCTCAZs and the scale of employment in related industries, thus exhibiting a strong correlation with NNCTCAZ distribution. Sichuan, Shandong, Zhejiang, Jiangsu, Shanghai, Beijing, Chongqing and other provinces and municipalities directly under the central government are densely populated, with abundant labour resources in the tertiary industry. These advantages offer a broad tourist source and labour market for the formation and development of local NNCTCAZs. For instance, the Wanshou Palace Historical and Cultural Block in Nanchang City, Jiangxi Province, is a large, multifaceted block that integrates fashion, shopping, catering and entertainment, social leisure and cultural tourism. The Wanshou Palace Historical and Cultural Block is located in the heart of the city, surrounded by shopping malls and close to the Bayi Uprising Memorial Hall, Bayi Square and other thriving areas, resulting in a large passenger flow, many service employees and strong serviceability. This environment is conducive to the development of the nighttime cultural tourism economy.

The spatial layout of NNCTCAZs is also affected by the level of tourism industry development, with an average q value of 0.784. The evaluation of the night tourism industry was influenced by several factors, and the evaluation indices showing the strongest correlation are as follows: total tourism revenue, with a value of 0.795; total number of tourists, with a value of 0.723; the national resort, with a value of 0.719; number of 5A scenic spots, with a value of 0.674 and number of star-rated hotels, with a value of 0.599. The total tourism revenue and the total tourist times in a region considerably impacted the spatial distribution of NNCTCAZs, in addition to tourism resources, which had a strong impact on the NNCTCAZs. Of the 243 NNCTCAZs, 19 (accounting for 7.82 %) are focused explicitly on tourist attractions, most of which are centred around urban sightseeing, cultural tourism and fashion business travel experiences.

Jingdezhen Taoyangli Royal Kiln Scenic Spot is in the heart of the old city of Jingdezhen and is classified as a national 4A-level scenic spot. This attraction comprises several key elements, including the Imperial Kiln Factory site, which is recognised as a national cultural relics protection unit, as well as the Royal Kiln Museum, traditional folk houses, porcelain shops, a kiln-making community and other historical and cultural relics related to the ceramic industry. These features combine to create diverse ‘ceramic culture, leisure and entertainment’ experiences for visitors.

Compared to traditional scenic spots, the tourist resort area has multiple consumption attributes, such as cultural experience, leisure vacation, tourism trade and fashion shopping. The area is also a suitable scenic spot for the development of NNCTCAZs. For instance, Guangzhou Chimelong Tourist Resort has four top theme parks and four theme hotels. The resort combines tourist attractions, hotels, catering, entertainment and recreation.

The formation of NNCTCAZs in these areas can be attributed to three factors. First, tourism resources are the basis for product development in NNCTCAZs. Second, the scenic spot has noticeable brand advantages and a large tourist base, with the innate advantage of developing into an NNCTCAZ. Jinji Lake Scenic Spot in Suzhou City, Jiangsu Province; Longmen Grottoes in Luoyang City, Henan Province; the Bund Scenic Spot in Huangpu District of Shanghai and the Princess Wencheng Tibetan Cultural Garden in Lhasa, Tibet, are well-known tourist attractions in China. With the increasing number of tourists staying overnight, a solid foundation for developing a cluster area focused on cultural tourism during the night has been established. Third, tourist attractions are the primary location for various tourism-related activities, featuring a diverse range of commercial establishments and well-developed tourism infrastructure, laying the essential groundwork for establishing a cultural tourism hub focused on nighttime consumption. For instance, Bund Scenic Area in Huangpu District of Shanghai integrates businesses such as night food, night tours, night shopping and night accommodation with perfect tourism infrastructure, promoting a high-level NNCTCAZ.

Based on individuals’ physiological characteristics and sleep patterns, nighttime tourism tends to revolve around leisure and entertainment activities. Accordingly, food and leisure services are in high demand, and tourists typically prefer to stay in accommodations located close to their intended destinations [38]. Therefore, various lodging facilities play a crucial role as supporting amenities. The spatial distribution of star hotels and other service facilities also has a significant impact on the formation and development of NNCTCAZs.

**Fig. 6:**
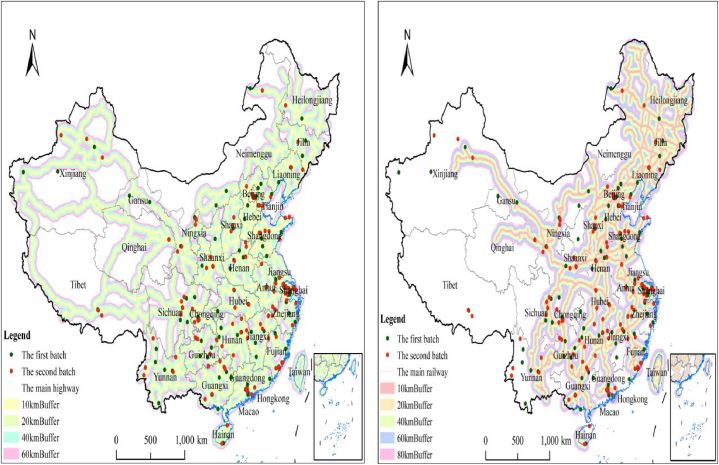
(a) Spatial relationship between NNCTCAZs and main highways. (b) Spatial relationship between NNCTCAZs and main railways.

The buffer analysis function module in ArcGIS 10.5 was used to determine the effect of traffic conditions on the spatial distribution of NNCTCAZs. In this method, buffer zones of varying distances (0–10, 20, 40 and 60 km) were established around major railways and highways throughout China. As shown in Fig. 5, most NNCTCAZs are concentrated within buffer zones of 0–10, 20 and 40 km around major railways and highways. The analysis of the intersection between points (NNCTCAZs) and lines (highway or railway) revealed that 243 NNCTCAZs are located in the 0–10-km buffer zone, constituting 80.66 %, and in the 40-km buffer zone, constituting 97.94 % of major highways (Fig. 6a). Furthermore, 243 NNCTCAZs are located in the 0–10-km buffer zone, constituting 60.08 %, and in the 40-km buffer zone, constituting 82.30 % of major railways (Fig. 6b). The significant ‘along the road’ distribution pattern observed in NNCTCAZs provides conditions conducive to attracting tourists and achieving sustainable development in these areas.

The average q value for the influence of traffic conditions on the spatial layout of the NNCTCAZ is 0.693. The q values for the influence of annual passenger traffic volume and highway density are 0.810 and 0.577, respectively. The traffic network connects the tourist destination and tourist source markets. Moreover, the traffic network significantly affects the radiation range and passenger flow of NNCTCAZs. Consumers select nighttime recreation and consumption destinations close to their accommodations that have diverse transportation options, low transportation costs and strong transportation capacity. Thus, good traffic conditions serve as a basis for the formation and development of NNCTCAZs, consistent with the buffer zone analysis findings.

The cultural environment also affects the spatial layout of NNCTCAZs, with an average q value of 0.426. The q values for the influence of the number of cultural performance groups and the number of national intangible cultural heritage sites are 0.475 and 0.376, respectively. Cultural undertakings significantly influence the spatial heterogeneity of the NNCTCAZs. Tourism and folk performances are relevant components of nighttime tourism products and are closely linked to local art groups and cultural resources. In regions where cultural undertakings are thriving, the creation and production of fine art products are more active. This can effectively transform local cultural resources into cultural performing arts products, thereby providing a more diverse range of nighttime tourism products for the region and promoting the formation and development of NNCTCAZs.

The most typical example is Taishan, where the core area of Shandong Taishan Xiucheng Performing Arts is located at the foot of Taishan, covering five major industries, namely, cultural performing arts, immersive entertainment, Taishan's intangible cultural heritage food street, boutique lodging and national trendy culture and creativity. These five core elements form a destination-type scenic spot, with performing arts as the core industry, integrating acting, entertainment and folklore, and creating another ecological chain of cultural tourism integration of ‘climbing Taishan + visiting Xiucheng’. Nighttime live performing arts products are essential for enriching the nighttime tourism experience and attracting visitors to stay overnight and extend their stays. This can stimulate the development of the entire tourism industry chain, including accommodations, dining, shopping and entertainment. Promoting the consumption of night tourism products can upgrade the entire industry, creating a more sustainable and prosperous night tourism destination.

Cultural resources also influence the spatial distribution of NNCTCAZs. China has an impressive cultural heritage, with 3610 national intangible cultural heritage items and 43 items listed in the UNESCO Intangible Cultural Heritage List, making it the country with the most listed items globally. These intangible cultural heritage items are an abundant source of local art creation, providing a valuable ‘raw material’ for the design of NNCTCAZs. Cultural diversity is conducive to the differentiated development of NNCTCAZs. However, the low impact of cultural resources indicates that the current role of cultural resources in promoting the creation and development of NNCTCAZs has not been completely realised.

## 6 Discussion

Previous studies on the spatial distribution of the tourism and leisure industry [23], National Geoparks [24], and Beautiful Leisure Villages [25] have primarily focused on macro factors such as the economy, population, geography, and transportation, with less attention given to subdividing into secondary indicators. In this study, we build upon existing research by identifying five major influencing factors and sixteen secondary indicators, providing a more comprehensive analysis. This approach not only enables us to quantify the specific impact of each secondary indicator but also facilitates the synthesis of results related to macro factors. The NNCTCAZ is also influenced by population, economic, and traffic factors. This is consistent with the results of previous studies [[23], [24], [25]], but the results of the influence of the proportion of the tertiary industry in the level of regional economic development on the q value are inconsistent with the results of a previous study [10], which may be attributed to the different geographic scopes of the study and the need for more studies to verify the results.

In the present study, the nighttime lighting index (q = 0.101) has a relatively weak influence on the spatial layout of the nighttime cultural and tourism consumption agglomeration area, which is consistent with the results of the study by Luo et al. There is a difference in the results of the study with references [1,49], and the study by Ref. [49] shows that the degree of centralisation and spatial agglomeration coupling between the nighttime lighting index and the tourism economy is higher in China, such as in East China, North China and other areas. At the same time, when verifying the influence factors of traffic, this study not only verified the highway density by using the ground detector, and the mean value of the influence of passenger transport volume was 0.693, but also verified the characteristics of the distribution of NNCTCAZs ‘along the road’ by using the ArcGIS 10.5 buffer analysis function module, which is consistent with the results of the study in Ref. [1].

In the existing studies on the spatial distribution characteristics of nighttime tourism resources, little attention has been paid to the influence of the cultural environment [1,50]. Currently, the high demand for nighttime cultural tourism consumer products exceeds the available supply, suggesting that the scarcity of production factors is a key factor affecting the development level of national cultural tourism zones. Currently, only 2.47 % of cultural tourism resources are used for performance purposes, which is similar to a previous study [51]. Peng and Chang stated that China's night tourism is not well targeted, supporting services cannot meet the needs of tourists and the main consumer group of night tourism is young people. The lack of cultural, leisure, athletic and sports products makes it difficult to attract young people, and thus it is not easy to form a unique night tourism brand. Therefore, the capitalisation of cultural resources is particularly relevant for the creation and development of NNCTCAZs. Against the background of the nation's vigorous construction of NNCTCAZs, we must pay urgent attention to the insufficient excavation of local cultural connotations and the insufficient supply of high-quality cultural products in the process of the creation and development of NNCTCAZs to avoid the homogeneous development of nighttime cultural tourism consumption clusters. The construction of NNCTCAZs is a relevant way for tourist destinations to retain overnight tourists and expand the scale of NNCTCAZs.

### 6.1 Theoretical implications

First, the results of this study expand the application of spatial structure theory in night tourism, especially NNCTCAZs. Based on spatial structure theory, the GIS spatial statistical analysis method is used to derive the spatial distribution characteristics of NNCTCAZs and the factors affecting their distribution, providing another cognition for night tourism NNCTCAZs.

Second, combining the characteristics of NNCTCAZs and the relevant characteristics of nighttime tourism and referring to the previous research, we constructed an indicator system affecting NNCTCAZ development, which made up for the lack of spatial exploration of the NNCTCAZs. The indicator system can promote the development of nighttime tourism research, provide a reference for the practical and reasonable development of the spatial distribution of NNCTCAZs and at the same time provide insights into the development of the nighttime economies of other countries. Overall, the present study provides new ideas for the development of nighttime tourism.

### 6.2 Practical implications

To promote the development of NNCTCAZs and improve the vitality of urban tourism, this study offers the following recommendations.

First, it is essential to promote the upgrading of the regional economic structure, vigorously develop the local economy, focus on improving the regional economic development level and residents’ quality of life, improve public support facilities and strengthen cross-border linkages among the cultural, tourism and sports sectors to lay a solid foundation for the development of regional NNCTCAZs.

Second, efforts should be made to expand the supply of leisure services; promote the clustering of ‘food, tourism, shopping, entertainment, sports, exhibitions, performances’ and other industries; tap into the potential of scenic spots and historic districts; develop nighttime cultural tourism projects; strengthen support for nighttime tourism and performing arts; accelerate the cultivation of various types of special nighttime cultural tourism projects and enhance the charm of nighttime tourism culture and art.

Third, we must establish several leading cultural tourism enterprises, encourage local cultural performing groups to conduct cultural and artistic creations, deeply excavate local characteristic cultural resources, such as regional culture, history and humanities and introduce unique regional cultural elements to develop characteristic tourism projects, deepen the impression of the ‘tourism business card’ of the cities where scenic spots and attractions are located and enhance the cultural and artistic charms of night tours. This approach can create a nighttime culture and tourism consumption agglomeration area brand, capitalise on cultural resources, bring new vitality to the city and help achieve dual economic and social benefits.

Fourth, the supply of nighttime transport services should be increased, nighttime running hours of public transport at a tangible time should be extended, more diversified modes of transport access should be provided and the accessibility of nighttime cultural and tourism consumption agglomeration areas must be improved.

Fifth, measures must be adopted to strengthen marketing promotion and brand building and continue to promote the quality and upgrading of the clusters to ensure that the clusters become the priority of tourists and residents for nighttime consumption.

### 6.3 Research limitations and future research directions

This study mainly analyses the spatial distribution of all 243 NNCTCAZs in China without including the local-level cultural and tourism consumption agglomerations in each city, suggesting some limitations in the sample size. Another limitation of this study pertains to the three indicators in terms of the development level of the tourism industry: (1) selection of scenic spots: due to the difficulty of considering 3A and 4A scenic spots in each province and city because of their abundant distribution, only 5A scenic spots are selected for analysis; (2) selection of resorts: only national-level tourist resorts are analysed while ignoring provincial resorts; (3) choice of star-rated hotels as the embodiment of reception capacity: although only star-rated hotels are selected, tourists often select specialty small restaurants, bars, B&Bs and other tourism reception facilities. Therefore, future research on provincial and municipal night cultural and tourism consumption agglomeration areas should consider 3A and 4A scenic spots, provincial tourism resorts, the number of beds and the number of restaurants as indicators to test their influence and conduct a comparative analysis.

## 7 Conclusion

Using ArcGIS software, this study uses an integrated approach combining the nearest index, geographic concentration index, Lorentz curve, imbalance index, nuclear density estimation and geographical detector methods to explore the spatial distribution characteristics of NNCTCAZs and the influencing factors.

The main conclusions are outlined as follows. First, the spatial distribution of NNCTCAZs in China is unbalanced, with an overall distribution characterised by ‘a strong east, a thriving west and a progressive centre, with small agglomerations and large dispersions’ pattern. At the level of provinces, autonomous regions and municipalities directly under the central government, NNCTCAZs are mainly concentrated in Sichuan, Shanghai, Jiangsu, Zhejiang, Shandong, Chongqing, Beijing, Fujian, Jiangxi, Guangdong and the Guangxi Zhuang Autonomous Region. These 11 provinces, municipalities and autonomous regions constitute 52.68 % of such areas in China. Furthermore, 41.56 % of the NNCTCAZs are located in the eastern region, whereas 23.05 % and 35.39 % are in the central and western regions, respectively, suggesting that the eastern region has a higher concentration of NNCTCAZs than the central and western regions. The distribution of NNCTCAAZs varies among the seven geographical regions, with the highest distribution in the east region at 32.10 % and the lowest in the northeast region at 4.53 %. Second, the spatial distribution of NNCTCAZs is directional, forming ‘a dense concentration east, a sparse west and a single-core, multicentre’ clustering pattern. NNCTCAZs are mainly distributed in urban areas with a good economic foundation, a dense population, good traffic conditions, a developed tourism industry and around famous tourist attractions. The ‘core-edge’ structure feature is relatively noticeable. Third, the spatial distribution of NNCTCAZs is comprehensively influenced by a combination of economic, social and other factors. Specifically, the level of social development, the tourism industry and regional GDP are the core factors affecting the spatial layout of NNCTCAZs. The level of development of the traffic conditions is a relevant influencing factor, and the cultural environment and economic development level are general factors influencing the spatial layout of NNCTCAZs. The decision-making basis can be provided by the results of the reasonable layout of NNCTCAZ, and the direction can be provided to night tourism operators and enterprises to develop characteristic cultural tourism products.

## Funding

This work was supported by the Nanchang federation of Social Science Association (Grant numbers [YJ202210]), Humanities and Social Sciences Programme of Higher Education Institutions in Jiangxi Province (Grant numbers [MKS18103])，Educational Science Planning Project for Higher Education Institutions in Jiangxi Province (Grant numbers [22YB293]) and Jiangxi Province Department of Education Science and Technology Program key projects (Grant numbers [GJJ212518])

## Data availability statement

The datasets used and/or analysed during the current study are available on request.

## CRediT authorship contribution statement

**Shanmei Xiong:** Formal analysis, Data curation, Conceptualization. **Hui Wang:** Writing – review & editing. **Zhenwei Liao:** Formal analysis. **Rahmat Hashim:** Visualization, Validation, Supervision.

## Declaration of competing interest

The authors declare that they have no known competing financial interests or personal relationships that could have appeared to influence the work reported in this paper.
